# Family medicine in post-communist Europe needs a boost. Exploring the position of family medicine in healthcare systems of Central and Eastern Europe and Russia

**DOI:** 10.1186/1471-2296-13-15

**Published:** 2012-03-12

**Authors:** Marek Oleszczyk, Igor Švab, Bohumil Seifert, Anna Krztoń-Królewiecka, Adam Windak

**Affiliations:** 1 Department of Family Medicine, Chair of Internal Medicine and Gerontology, Jagiellonian University Medical College in Krakow, Krakow, Poland; 2 Department of Family Medicine, Faculty of Medicine, University of Ljubljana, Ljubljana, Slovenia; 3 Department of General Practice, First Medical Faculty, Charles University in Prague, Prague, Czech Republic

## Abstract

**Background:**

The countries of Central and Eastern Europe have experienced a lot of changes at the end of the 20th century, including changes in the health care systems and especially in primary care. The aim of this paper is to systematically assess the position of family medicine in these countries, using the same methodology within all the countries.

**Methods:**

A key informants survey in 11 Central and Eastern European countries and Russia using a questionnaire developed on the basis of systematic literature review.

**Results:**

Formally, family medicine is accepted as a specialty in all the countries, although the levels of its implementation vary across the countries and the differences are important. In most countries, solo practice is the most predominant organisational form of family medicine. Family medicine is just one of many medical specialties (e.g. paediatrics and gynaecology) in primary health care. Full introduction of family medicine was successful only in Estonia.

**Conclusions:**

Some of the unification of the systems may have been the result of the EU request for adequate training that has pushed the policies towards higher standards of training for family medicine. The initial enthusiasm of implementing family medicine has decreased because there was no initiative that would support this movement. Internal and external stimuli might be needed to continue transition process.

## Background

Central and Eastern Europe (CEE) experienced dramatic changes by the end of the 20th century. The changes were marked by the fall of communist ideology and regimes in late 1980s and early 1990s. After that, the former communist countries took different ways of social and political transformation. Some of the countries became members of the European Union, while the others struggled with economic and political instability.

The expansion of the European Union had an important effect on CEE countries. Although organization of health care is left to the member states, Europe is moving towards common standards in health care provision [1].

The health care systems of CEE countries were not identical before transformation and they roughly fall within two categories: the systems based on the Soviet Semashko model [2-10] and the system of former Yugoslavia [11,12]. The changes were far-reaching in all areas of the health care system, especially in primary care. In the post-Semashko countries, the institution of primary care and general practice/family medicine (in the Wonca definition understanding) needed to be implemented from the beginning, which was often assisted by international support [13].

It is remarkable that these drastic changes were not often reported in scientific journals. Most of the reports were descriptive and almost narrative. There were very few studies using common tools to describe the situation [6,14-17].

The Family Medicine After Transformation in Middle and Eastern Europe (FATMEE) study (initiated by Wonca Europe) aimed to explore the position of family medicine in the health care systems as well as the status of academic development of the discipline in the studied countries. The aim of this paper is to explore:

• the current role and perspectives of family medicine/general practice in healthcare systems of CEE countries and Russia

• the mechanisms of quality assurance in family medicine/general practice in this region.

## Methods

### Instrument

**Figure 1:**
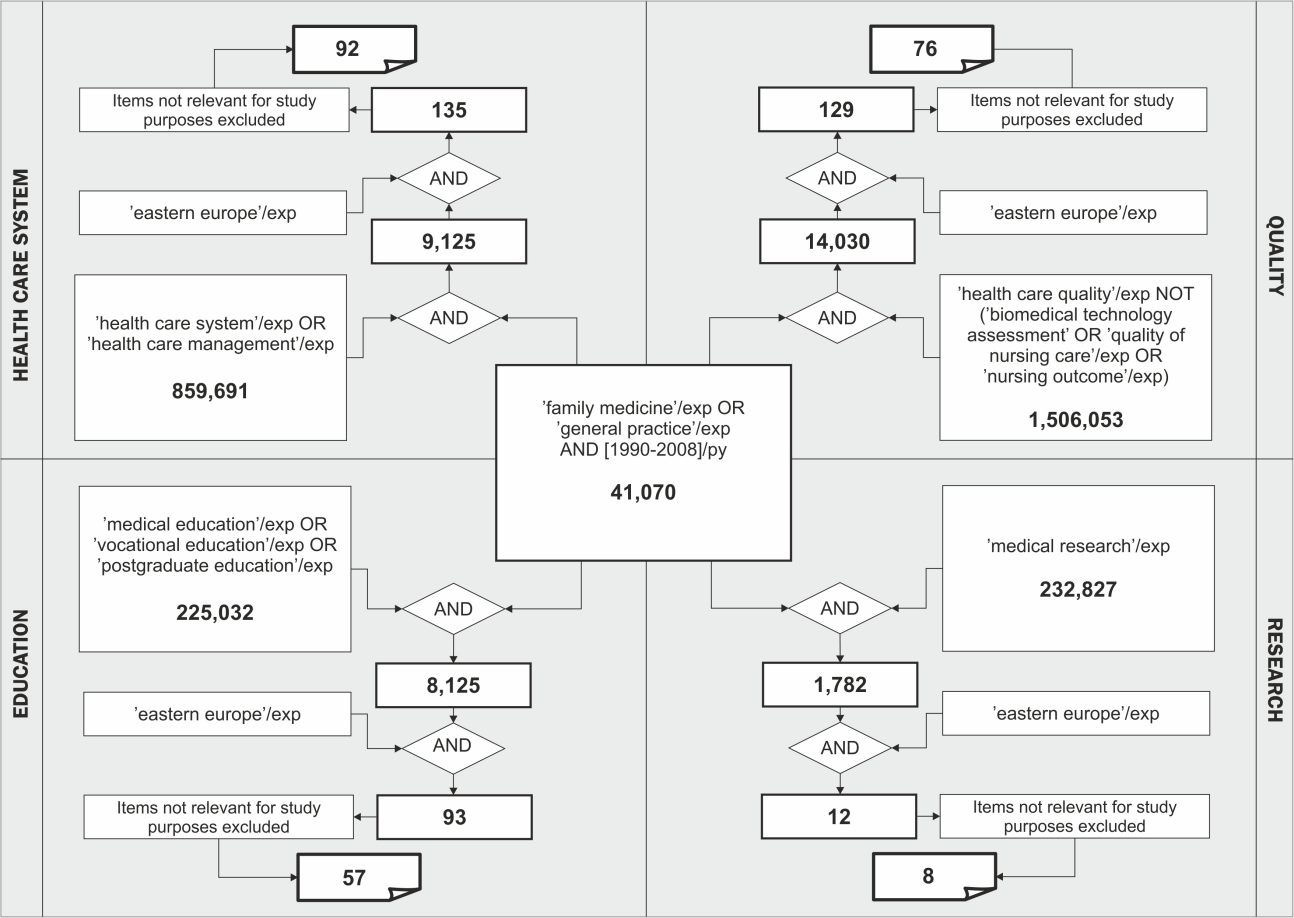
**Embase (including Medline) systematic literature search strategy**.

In the first step, a systematic review of literature was conducted in order to identify indicators that would describe the position of family medicine in CEE on a country level and that would be comparable internationally. Ovid Embase resources (covering MedLine records) were searched. Combinations of relevant Emtree terms were used. The review was conducted at the very beginning of 2009; we searched for the papers published in period 1990-2008. The details and results of searching strategy are presented in Figure 1.

Finally 92 records in area of 'Health Care System', 76 records in area of 'Quality', 57 in area of 'Education' and 8 in area of 'Research' in Eastern Europe region were identified. Based on the review a set of indicators was proposed to the study group. The indicators were agreed by the study group through a consensus that took place over an e-mail mediated process that was led by the project coordinators (AW and MO). The indicators were then transformed into sets of questions, which formed a basis for the questionnaire. The draft version of the questionnaire was piloted in Czech Republic, Slovenia and Poland by staff members of university family medicine departments. After revision of their comments the final version of the questionnaire, consisting of 57 questions was developed. 34 questions concerned the position of family medicine in a healthcare system.

### Countries and informants

We aimed to gather the data from the largest possible number of countries of Central and Eastern Europe. We used the key-informant strategy for gathering the information. The informants were selected from a list of key representatives of the Wonca Europe member countries. They were supposed to be individuals involved in primary care development and/or teaching with the position of local expert in the field of primary care and/or family medicine/general practice. They were selected from different institutions. In each country two independent informants, suggested by Wonca Europe national representatives, were recruited. Through all the study time the identity of the in-country counterpart was not revealed to each other. Because of non-experimental design of the study, ethical approval had not to be sought.

### Data collection

For each of the key informants a personal invitational letter was sent. Those who agreed and confirmed their participation received an e-mail with direct link to the on-line questionnaire. The internet-based tool Survey Monkey ^® ^was used for data collection.

The strategy involved two rounds of data collection: in the first round, the data were collected centrally. The results were analysed by two independent researchers. They have labelled all the answers where agreement was not obvious or the data were missing. In these cases, the disagreements were identified and commented. This information was then sent to the key informants again for the second round.

The informants were asked to review all "missing" and "disagreed" issues and challenged to explain their previously given opinion in cases of differences. They were also encouraged to make open-ended comments when needed. The informants were also asked to seek reliable resources (e.g. official documents, reports, papers), that could clarify the disagreements and provide the validation for one of the statements. The answers were then again analysed by two independent researchers. For the final analysis only the agreed and validated issues were included.

**Figure 2:**
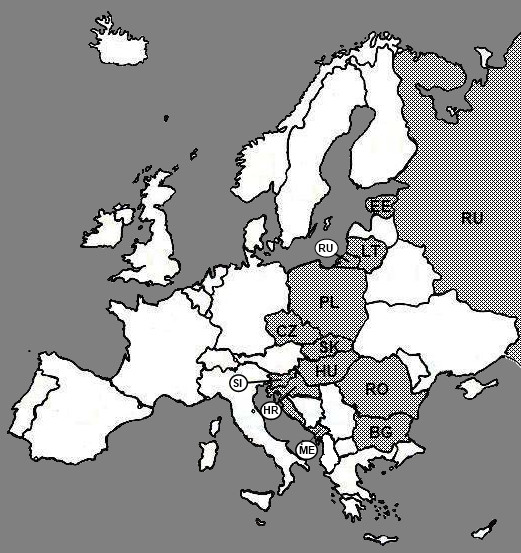
**Countries included in the study**.

11 countries of CEE and Russia were finally included for the study. Amongst European Union member states we failed to recruit key informants only from Latvia. The rest of EU-member states studied were Bulgaria (BG), the Czech Republic (CZ), Estonia (EE), Hungary (HU), Lithuania (LT), Poland (PL), Romania (RO), Slovakia (SK) and Slovenia (SI). The non-EU member states studied were Croatia (HR), Montenegro (ME) and Russia (RU) Figure 2.

The first round of data collection lasted from June through August 2010. The second--from November 2010 through March 2011.

## Results

### Informants

The informants had extensive professional experience (mean 26,3 years since graduation min. 9 max. 40 years); 83% were physicians, while the rest were other health care professionals; 25% had a title of a professor; 25% post-PhD or associate professor; 12,5% - PhD. 19 (nearly 80%) of them were family physicians/general practitioners. Majority (83%) worked in primary health care, 62% were employee at the University/Medical School.

### Position in the healthcare system

The results show that position of family medicine/general practice is regulated by law at various levels in all 12 countries (act of parliament or ministerial decree). In all the countries family medicine/general practice is recognized as a separate specialty, theoretically on the same basis as other medical disciplines. The strategic documents that would define the development of primary care exist in Bulgaria, Estonia, Romania, Slovakia, Slovenia, Croatia, and Montenegro. All the countries, except Romania, have their competencies legally described, but the agencies that are responsible for that vary. In Bulgaria, Poland, Slovakia, Montenegro and Russia the government describes the competences of family physicians/general practitioners. In the Czech Republic and (partially) in Poland health insurance companies are responsible for it while in Slovenia this is a task of the college of physicians. In Hungary, Estonia, Lithuania and Croatia the experts could not agree in this issue.

The organization of care provided by family physicians/general practitioners is the responsibility of central government in Bulgaria, Romania, Slovakia, Croatia, Montenegro, Russia, and recently (since 2011) in Estonia. The strong regulative role of insurance companies, heavily influencing organization and provision of care is reported by respondents in Bulgaria, the Czech Republic, Estonia, Poland, Romania and Montenegro. Some of the organizational competences are dedicated to local authorities in Bulgaria, the Czech Republic, Lithuania, Poland, Slovakia, Croatia and Russia. No clear and consistent data on organizational responsibilities were obtained in Hungary and Slovenia.

In most of the countries, family physician/general practitioner is not an exclusive physician to provide primary care services. Such situation exists only in Estonia. In Bulgaria and Poland paediatricians and internists are allowed to work in primary care but there is a deadline by which they would have to be re-trained in family medicine to remain primary health care service providers (Poland in 2017, Bulgaria in 2015). Paediatricians are also involved in primary care in the Czech Republic, Slovakia and Slovenia. In Russia all medical specialists are directly available in polyclinics. Detailed comparison of the specialties involved in delivery of primary care is presented in Table 1.

**Table 1:** Specialties involved in primary health care in different countries

	FDs/GPs	Internists	Paediatricians	Obstetricians & Gynecologists	Other specialists	Physicians w/o any specialty
**EU Countries**						

BG	✓	✓	✓			

CZ	✓		✓			

EE	✓					

HU	✓		✓			✓

LT	✓	✓	✓	✓	✓	

PL	✓	✓	✓			

RO	✓					✓

SK	✓		✓	✓		

SI	✓		✓			

**Non-EU Countries**						

HR	✓	✓	✓	✓	✓	✓

ME	✓	✓	✓	✓	✓	✓

RU	✓	✓	✓	✓		

Although exceptions exist in some of the countries, it is not possible to work independently without specific training. Doctors without specific training can work in primary care in Hungary (no longer than 5 years), Romania, Croatia and Montenegro.

#### Working conditions

In 5 out of 12 countries family physicians/general practitioners have a position of (mostly) independent contractors (Bulgaria, the Czech Republic, Estonia, Hungary, Romania and Slovakia) while in the others (Lithuania, Poland, Slovenia and Croatia) they can choose either to be state-employees or independent contractors. In Montenegro they are mostly state-employee. Only in Russia physicians are exclusively state-employees.

#### Organisation of care

**Figure 3:**
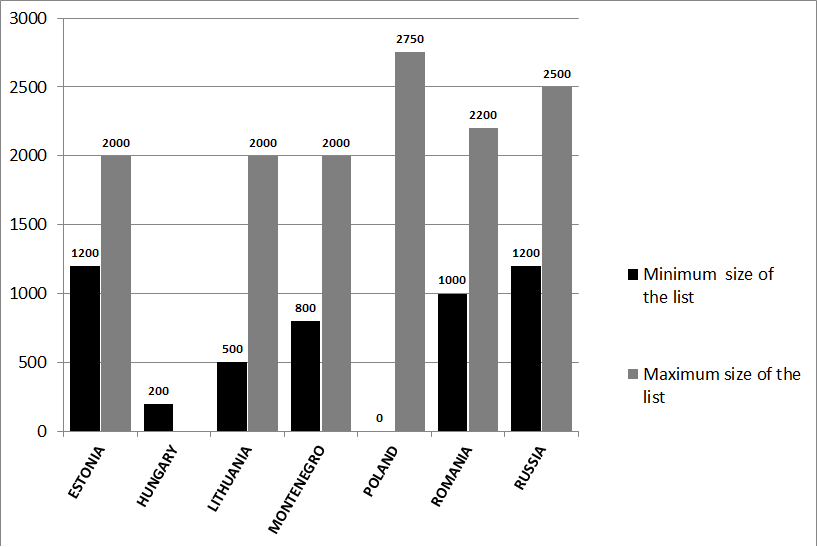
**Minimal and/or maximal patients' list size**.

In all study countries family physicians/general practitioners have their personal list of patients. In seven countries (see Figure 3) the list size is regulated but those regulations might be just formal (e.g. most of the Hungarian doctors--contractors of municipal authorities--are obliged to have larger than formal minimal list of 200 patients). There was no agreement on this matter in Croatia.

In some countries regulations exist to maintain desirable size of the patient list. In Estonia, Hungary, Slovenia and Croatia the decreased capitation fee is applied to discourage excessive patient lists. In Russia, although regulations are present, larger patient lists are common due to a shortage of physicians.

Various practice organisational arrangements are present in the studied countries. However in majority of the studied countries, solo practices are the most predominant form of organisation of services. Leading organisational forms of practices are presented in Table 2.

**Table 2:** Organisational forms of practices in the studied countries

	Single-handed	Group practice of 2 or 3 FD/GPs	Health centers with many FD/GPs	Health centers with FD/GPs and other specialists
**EU Countries**				

BG	✓			

CZ	✓			

EE	✓	✓		

HU	✓			

LT	✓ (rural area)			✓(cities)

PL	✓	✓		

RO	✓			

SK	✓			

SI			✓	✓

**Non-EU Countries**				

HR	✓			

ME				✓

RU	✓ (in some remote locations)			✓

**Figure 4:**
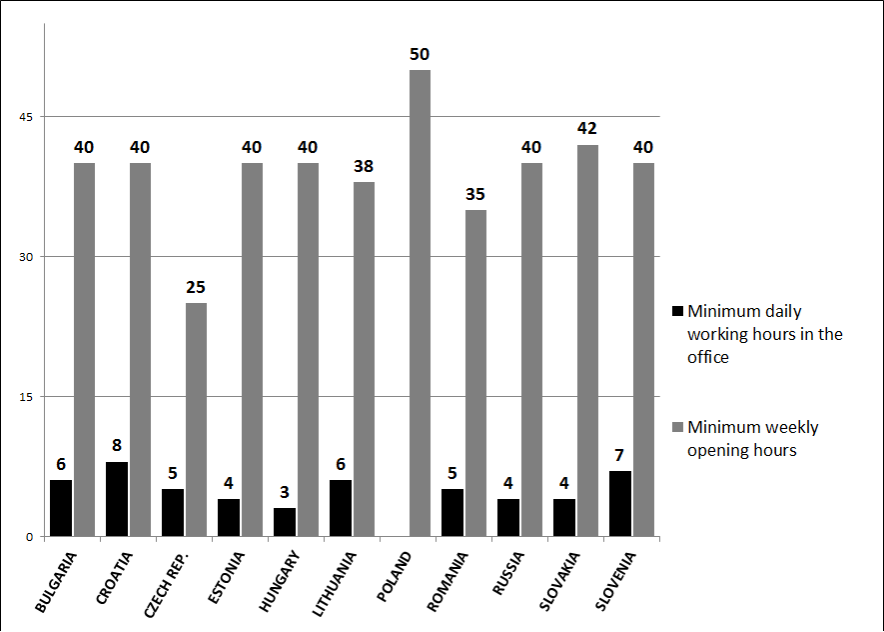
**Minimal weekly-opening and daily-working in office hours**.

Opening hours (availability of services) are regulated in all countries. The working time differs and is presented in Figure 4. The average minimum working hours are around 40 hours/week, but in case of the Czech Republic (25 hours/week) and Poland (50 hours/week) the difference is 100%. Physician's minimum daily availability for patients also differs--from 3 hours/day in Hungary to 8 hours/day in Croatia, while in Poland the situation is not clearly regulated.

#### Financing

Financing of family physicians/general practitioners' services in all countries is based on mixed systems. The major part of this payment is usually capitation fee. The details about the payment methods are presented in Table 3.

**Table 3:** Payment methods for services

	Capitation	Fee for service	Fee for preventive activities	Other
**EU Countries**				

BG	✓	✓	✓	Bonus for physicians working in remote and disadvantageous practices

CZ	✓	✓		Capitation (70%), fee for services (25%) regulative fee (co-payment) (5%)

EE	✓	✓	✓	Quality bonus Remote practices (> 40 km from hospital), budget for diagnostic procedures

HU	✓			Rural area bonus, age wages, population size wages, quality bonuses

LT	✓	✓	✓	

PL	✓		✓	

RO	✓			

SK	✓(90%)	✓(10%)		Fee-for-service is allowed only when physician performs preventive examination, voluntary vaccination and on the home visit (those services are paid by patient out of pocket)

SI	✓(50%)	✓(50%)		

**Non-EU Countries**				

HR	✓(80%)	✓(10%)	✓(10%)	Preventive activities are paid when goals are reached

ME	✓		✓	Bonus for physician depending on duration of employment

RU	✓	✓ (in private clinics)		Additional payment from state programs' budget (e.g. tuberculosis, dermato-venerology)

#### Gate-keeping

The gate-keeping role of the family physicians/general practitioners varies. The 'total gate-keeping' formally exists only in Croatia and Montenegro, while in the remaining countries the gate-keeping is partial and several specialists are directly accessible without prior referrals. In most of the study countries patients can also directly access specialist care when they pay out-of-pocket. There was no consensus in Romania regarding this matter. An overview of availability of specialty care through general insurance is shown in the Table 4.

**Table 4:** Specialists available without FD/GP's referrals

	Obstetrician Gynaecologist	Paediatrician	Internist	Ophthalmologist	ENT	Oncologist	Dermatologist	Surgeon	Dentist	Psychiatrist	Other
**EU Countries**											

BG	✓	✓							✓		

CZ	✓	✓	✓	✓	✓	✓		✓	✓	✓	

EE	✓			✓		✓	✓		✓	✓	

HU	✓	✓		✓	✓		✓	✓	✓	✓	pulmonologist

LT							✓(!)		✓	✓	

PL	✓			✓		✓			✓		

RO						✓			✓		

SK	✓			✓						✓	

SI	✓	✓									

**Non-EU Countries**											

HR	✓	✓									

ME	✓	✓							✓	✓	

RU	✓	✓	✓	✓	✓		✓(!)	✓(!)	✓	✓(!)	

!-no agreement, confirmed by only one key informant

#### Out-of-hours care

Family physicians/general practitioners are largely involved in emergency services, which is the case in Bulgaria, Hungary, Lithuania, Croatia, and Montenegro. In the Czech Republic, Estonia, Poland, Romania, and Slovakia those services are organised separately. In Slovenia and Russia various organisational arrangements are present.

#### Range of services

The range of provided services differs across the countries. In Table 5 opinions of key informants regarding the frequency of services performed by family physicians/general practitioners in daily practice are presented. In all of the studied countries family physicians/general practitioners provide office consultations and home visits. Regular check-up and assessment for social services is done by them in all countries except Russia. Family physicians/general practitioners make also phone consultations, although in Poland and Russia this type of consultation is not formally recognized. Group sessions for patients with specific problems were confirmed only in Montenegro, while in Estonia, Slovenia, Croatia and Russia respondents couldn't reach agreement on this matter and in the remaining countries they unanimously denied availability of this form of the consultation.

**Table 5:** Frequency of services provided by family physicians/general practitioners

	Curative care for children	Curative care for adults	Minor surgery	Prenatal and pregnancy care	Children surveillance and preventive care	Adults screening and preventive programs	Assessment for social services/insurance purposes
**EU Countries**							

BG	A	A	S	A	A	A	A

CZ	S	A	S	N	S	A	A

EE	A	A	S	S	A	A	A

HU	S	A	S		S	A	A

LT		A				A	A

PL	A	A	S	S	A	A	A

RO	A	A		A	A	S	S

SK	N	A	S	N	A	A	A

SI	S	A	S	N	S	A	A

**Non-EU Countries**							

HR	S	A	S	S	S	A	A

ME	S	A	S		S	A	S

RU	S	A	A	S	S	A	S

A-Always S-Sometimes N-Never

Empty boxes in areas where agreement could not be reached

### Quality assurance

Accreditation is usually done as an external and mandatory process of quality assurance. In all the countries there are regulations that determine the conditions for provision of services of family physicians/general practitioners and they are mainly regulated by the contract or legal rules. In Estonia independent contractors who voluntary join quality system get bonus to the contract. It is uncommon for family physicians/general practitioners to undergo additional voluntary accreditation procedure (e.g. ISO).

Traditional medical records in paper are still used in most of the countries (Bulgaria, the Czech Republic, Lithuania, Poland, Romania, Slovakia, Slovenia, Montenegro and Russia) although electronic medical records (EMR) are also used. In Hungary, Estonia and Croatia the process of introduction of EMR is more advanced. Family physicians/general practitioners in those countries use only this type of medical data management. In all countries (except the Czech Republic and Russia, where agreement could not be reached) computers are used for administrative purposes. In Bulgaria, Hungary, Estonia, Romania, Slovakia, Croatia and Montenegro nearly all physician use computers also for clinical purposes (EMR). Such use of computers is present in more than half of Czech practices and in minority of practices in Lithuania, Poland, Slovenia and Russia.

Guidelines developed specifically for the primary care doctor's purposes are common. The exceptions are Croatia, Slovakia and Montenegro. Peer-review groups (voluntary structures focused on continuous professional development) were present only in Poland, although minority of family physicians was involved. The reason for this could be lack of different than internal motivation factors.

## Discussion

### Summary of main findings

In all study countries position of family medicine/general practice is regulated and implemented by the law. Not all of the countries have clear strategy of family medicine future development. Family medicine is the exclusive medical specialty in primary care only in Estonia. Involvement of paediatricians and gynaecologists in primary care services is common. In some of the study countries physicians without any specialty are allowed to provide primary care services. Performance of curative and preventive procedures in adults, as well as assessment for administrative purposes is common in the study countries. Minor surgery procedures are rarely performed by family physicians. Financing of family/general practice is at most based on capitation fee and payment for preventive procedures. The size of the list of patients is regulated in some countries by the law. Financial mechanisms are also employed to limit the list size. The gate-keeping role is weak in most of the countries. Only in Russia family/general practitioners are exclusively state-employed. Service quality assurance is mostly based on legal and contract regulations. Other mechanisms are not common and based on internal motivation only.

### Strengths and limitations of the study

The strength of the study lies in the number of countries that we have managed to involve in it. However, still we were unable to gather data from some of the countries which are not members of Wonca Europe (e.g. Belarus, Kosovo). Nevertheless, we believe that this is one of the biggest collections of information from countries in Eastern Europe published so far [13,16,18]. The choice of methodology was based on the rationale that we were faced with complexity and diversity of conditions and we needed to explore data and legal documents, usually unavailable in English and unpublished in indexed international journals. We also needed interpretations of issues that are difficult to measure. We were faced with limited time and resources. We are well aware of the limitations of the key informants' strategy but we consider it as an appropriate choice of exploration of issues in our study. Such a strategy gave us possibility to have an insight in complex and broad area we were interested in. On the other hand it might lead to generalisations and approximations. Although we asked the informants to seek for 'hard data' whenever possible, their answers in the questionnaires should be considered as 'expert opinion'. We know that our findings could benefit from validation from quantitative studies that would require more time and resources. Still we think that our findings are based on reliable sources, that we have adhered strictly to the methodology and have tried to validate our information whenever possible.

### Contextualization of key findings

Formally family medicine/general practice exists as a legally recognized specialty in all countries. Practices and policies within many studied countries are often not in line with the formally accepted legislation. Family medicine is still considered as implementation of specialist services in primary care. In most of the study countries, family medicine is one of many medical specialties in primary care. This is especially case of the care for children and women, often provided by paediatricians and gynaecologists. Such situation might position family medicine as a general practice for adults only, especially in urban areas. These findings are consisted with the results reported also by other researchers [19].

The presence of specialists in family medicine/general practice is an important but not sufficient condition to make primary care efficient. Implementation of patient's right to free choice of physician [20], or privatisation of primary care can lead to quality improvement and increased patient's satisfaction [8,21,22].

On the other hand, a service payment method is crucial. Where capitation fee consists most of the physicians' income, doctors might be passive and avoiding cost-consuming procedures (e.g. minor surgery). Additional limiting of gate-keeping competences might lead to overload of secondary and tertiary care and involution towards *ancien régime*-policlinics system.

When the former policlinics were successfully disintegrated in nearly all of the countries of the previous Semashko system, they were replaced mainly by solo practices [2,17,23]. The situation regarding community oriented health centres in former Yugoslavia is somewhat different: they have often been replaced by solo practices (e.g. Croatia), but in some countries (e.g. Slovenia), they still form a backbone of primary care. The future will reveal whether solo practices would merge in group practices as it is in Western Europe.

Currently, only Estonia seems to have fully implemented family medicine model [24,25] - all the other countries are either slowly moving towards this direction or have stopped the transition. The rapid development of modern family medicine, experienced at the end of the 20th century is no longer taking place. This finding is consistent with the previous reports [26].

## Conclusions

We could not identify any common European standard of family medicine in the study countries. The countries within the EU do not differ remarkably from those outside the EU regarding policies and delivery of family medicine. The only difference is the implementation of postgraduate training in EU countries, which has not been implemented in the non-member countries yet. It looks like the initial enthusiasm of implementing family medicine in some of the study countries has waned. A lack of initiative from the EU to support this development might be one of the reasons.

Health care policy makers in CEE countries should re-think their visions of primary care. If family medicine-based model is still viable then multi-dimensional support is needed. If not, the training of new family physicians/general practitioners would be just *l'art pour l'art*.

## Abbreviations

Wonca: World Organization of National Colleges, Academies and Academic Associations of General Practitioners/Family Physicians; Wonca Europe: European regional branch of Wonca

## Competing interests

The authors declare no conflict of interest.

## Authors' contributions

MO contributed in conceptual work, study design, data collection, data analysis, drafting and revising of the manuscript. IS was involved in conceptual work, study design, data collection, drafting and revising of the manuscript. BS has contributed in conceptual work, study design, data collection and revising of the manuscript. AKK has been involved in data analysis and drafting the manuscript. AW had a substantial contribution in conception and design of the study, data collection and analysis, drafting and revising of the manuscript. All co-authors have given the final approval of the version to be published.

## Pre-publication history

The pre-publication history for this paper can be accessed here:

http://www.biomedcentral.com/1471-2296/13/15/prepub
